# Atalaphylline^1^

**DOI:** 10.1107/S1600536809051885

**Published:** 2009-12-09

**Authors:** Suchada Chantrapromma, Nawong Boonnak, Ibrahim Abdul Razak, Hoong-Kun Fun

**Affiliations:** a Crystal Materials Research Unit, Department of Chemistry, Faculty of Science, Prince of Songkla University, Hat-Yai, Songkhla 90112, Thailand; b X-ray Crystallography Unit, School of Physics, Universiti Sains Malaysia, 11800 USM, Penang, Malaysia

## Abstract

The title acridone alkaloid [systematic name: 1,3,5-trihydr­oxy-2,4-bis­(3-methyl­but-2-en­yl)acridin-9(10*H*)-one], C_23_H_25_NO_4_, known as atalaphylline, was isolated from *Atalantia monophylla* Corrêa, a mangrove plant. The mol­ecule contains three fused planar rings with an r.m.s. deviation of 0.026 (2) Å. Both 3-methyl­but-2-enyl substituents are in a (−)anticlinal conformation. An intra­molecular N—H⋯O hydrogen bond generates an *S*(5) ring motif, while an intra­molecular O—H⋯O hydrogen bond generates an *S*(6) ring motif. In the crystal structure, the mol­ecules are linked into screw chains along [010] by inter­molecular O—H⋯O hydrogen bonds. These chains are stacked along the *a* axis by π–π inter­actions with centroid–centroid distances of 3.6695 (13) and 3.6696 (13) Å.

## Related literature

For hydrogen-bond motifs, see Bernstein *et al.* (1995 ▶). For bond-length data, see: Allen *et al.* (1987 ▶). For details of acridone alkaloids and their biological activity, see: Basu & Basa (1972 ▶); Itoigawa *et al.* (2003 ▶); Kawaii *et al.* (1999*a* ▶,*b* ▶). For a related structure, see: Chukaew *et al.* (2007 ▶). For the stability of the temperature controller used in the data collection, see Cosier & Glazer, (1986 ▶).
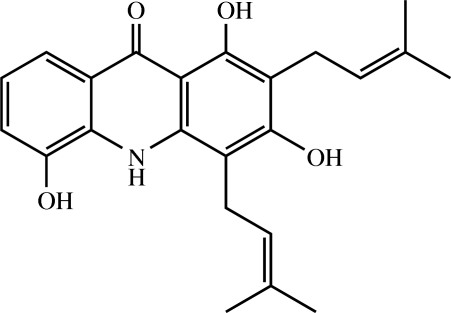


## Experimental

### 

#### Crystal data

C_23_H_25_NO_4_
*M* *_r_* = 379.44
Orthorhombic, 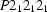

*a* = 5.0650 (1) Å
*b* = 15.0131 (4) Å
*c* = 24.5813 (5) Å
*V* = 1869.20 (7) Å^3^
*Z* = 4
Mo *K*α radiation
μ = 0.09 mm^−1^
*T* = 100 K
0.40 × 0.21 × 0.04 mm

#### Data collection

Bruker APEXII CCD area-detector diffractometer
Absorption correction: multi-scan (*SADABS*; Bruker, 2005 ▶) *T*_min_ = 0.964, *T*_max_ = 0.996
17852 measured reflections
3142 independent reflections
2525 reflections with *I* > 2σ(*I*)
*R*_int_ = 0.041

#### Refinement

*R*[*F* ^2^ > 2σ(*F* ^2^)] = 0.049
*wR*(*F* ^2^) = 0.118
*S* = 1.03
3142 reflections
257 parameters
H-atom parameters constrained
Δρ_max_ = 0.30 e Å^−3^
Δρ_min_ = −0.26 e Å^−3^

### 

Data collection: *APEX2* (Bruker, 2005 ▶); cell refinement: *SAINT* (Bruker, 2005 ▶); data reduction: *SAINT*; program(s) used to solve structure: *SHELXTL* (Sheldrick, 2008 ▶); program(s) used to refine structure: *SHELXTL*; molecular graphics: *SHELXTL*; software used to prepare material for publication: *SHELXTL* and *PLATON* (Spek, 2009 ▶).

## Supplementary Material

Crystal structure: contains datablocks global, I. DOI: 10.1107/S1600536809051885/sj2692sup1.cif

Structure factors: contains datablocks I. DOI: 10.1107/S1600536809051885/sj2692Isup2.hkl

Additional supplementary materials:  crystallographic information; 3D view; checkCIF report

## Figures and Tables

**Table 1:** Hydrogen-bond geometry (Å, °)

*D*—H⋯*A*	*D*—H	H⋯*A*	*D*⋯*A*	*D*—H⋯*A*
O1—H1*O*1⋯O2	0.82	1.82	2.554 (2)	149
O3—H1*O*3⋯O2^i^	0.82	1.93	2.752 (2)	175
N1—H1*N*1⋯O3	0.86	2.34	2.692 (3)	105

Symmetry code: (i) 
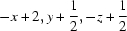
.

**Fig. 1.**
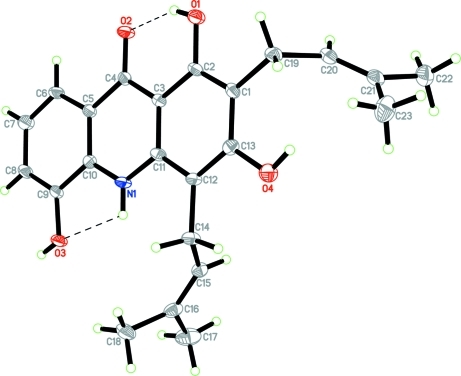
The asymmetric unit of (I), showing 50% probability displacement ellipsoids and the atomic numbering scheme. Intramolecular hydrogen bonds are shown as dashed lines.

**Fig. 2.**
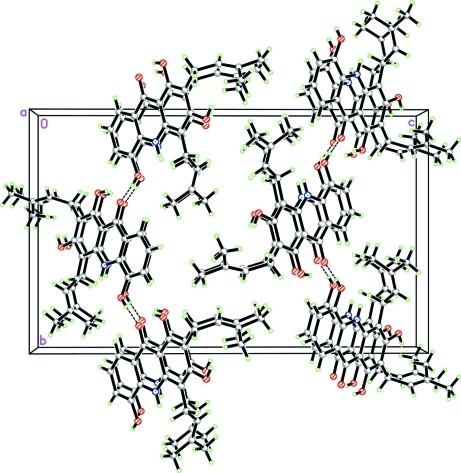
The crystal packing of (I) view along the a axis. Hydrogen bonds are drawn as dashed lines.

## supplementary crystallographic information

### Comment

Acridone alkaloids display a variety of biological activities such as antiproliferative (Kawii *et al.*, 1999*a*), induction of human promyelocytic leukemia cell (HL-60) differentiation (Kawii *et al.*, 1999*b*) and cancer chemopreventive activities (Itoigawa *et al.*, 2003). The title acridone alkaloid (I) known as atalaphylline (Basu & Basa, 1972) was isolated from *Atalantia monophylla* Corrêa, known locally in Thai as Manao Phi, a mangrove plant which was collected from Trang province in the southern part of  Thailand. We previously reported the crystal structure of *N*-methylataphyllinine, an acridone alkaloid which was isolated from the same plant (Chukaew *et al.*, 2007). As part of our research on the crystal structures of natural product compounds from Thai medicinal plants, the molecular and crystal structure of the title acridone alkaloid was investigated and is reported here.

The title molecule (Fig. 1) has a three-fused planar rings with an *r.m.s.* deviation of 0.026 (2) Å. The pyridine ring makes the dihedral angles of 1.82 (11) and 1.14 (11)° with the C1–C3/C11–C13 and C5–C10 benzene rings, respectively. All the three hydroxyl groups are co-planar with the attached benzene rings. All C atoms of each of the 3-methylbut-2-enyl substituents lie on the same plane with *r.m.s.*  deviations of 0.018 (2) and 0.004 (3)Å for the C14/C15/C16/C17/C18 and C19/C20/C21/C22/C23 planes, respectively. These two planes make dihedral angles of 88.55 (13) (for the 3-methylbut-2-enyl unit at atom C1) and 69.66 (13)° (for the 3-methylbut-2-enyl unit at atom C12) with the C1–C3/C11–C13 benzene ring. The torsion angles C2–C1–C19–C20 and C13–C12–C14–C15 are -103.0 (3) and -120.8 (2)°, indicating an (-)anti-clinal conformation of both the 3-methylbut-2-enyl units. An intramolecular N—H···O hydrogen bond generates an S(5) ring  while an intramolecular O—H···O hydrogen bond generates an S(6) ring motif (Bernstein *et al.*, 1995); these help to maintain the planarity of the acridone skeleton. The bond lengths in (I) are within normal ranges (Allen *et al.*, 1987) and comparable with those found in a related structure (Chukaew *et al.*, 2007).

In the crystal packing (Fig. 2), the molecules are linked into screw chains along the [0 1 0] direction by O3—H1O3···O2 hydrogen bonds (Table 1). These chains are stacked along the *a* axis (Fig. 2) by π···π interactions with distances *Cg*_1_···*Cg*_2_ = 3.6696 (13) Å and *Cg*_2_···*Cg*_3_ = 3.6695 (13) Å (symmetry codes for the interactions: 1 + *x*, *y*, *z* and -1 + *x*, *y*, *z* respectively); *Cg*_1_, *Cg*_2_ and *Cg*_3_ are the centroids of C3–C5/C10–C11/N1, C1–C3/C11–C13 and C5–C10 rings, respectively.

### Experimental

The air-dried and pulverized root of *A. monophylla* (6.0 kg) was exhaustively extracted with methylene chloride (2 × 20 l for one week) at room temperature. Removal of the solvent from the methylene chloride extract under reduced pressure gave a yellow viscous residue (52.5 g) which was subjected to quick column chromatography over silica gel using solvents of increasing polarity from n-hexane through EtOAc. The eluents were separated into 18 fractions (F1–F18) on the basis of TLC analysis. Fraction F12 (4.3 g) was further separated by quick column chromatography (QCC) with a gradient of acetone-hexane to afford 6 subfractions (12 A-12 F). Subfraction 12 C (385.0 mg) was further purified by QCC with a gradient of acetone-hexane to give the title compound (22.0 mg). Brown plate-shaped single crystals of the title compound suitable for X-ray structure determination were recrystallized from CHCl_3_/CH_3_OH (9:1, *v*/*v*) after several days, m.p. 518–520 K.

### Refinement

All H atoms were positioned geometrically and allowed to ride on their parent atoms, with d(O—H) = 0.82 Å, d(N—H) = 0.86 Å and d(C—H) = 0.93 Å for aromatic and CH, 0.97 for CH_2_ and 0.96 Å for CH_3_ atoms. The *U*_iso_ values were constrained to be 1.5*U*_eq_ of the carrier atom for hydroxy and methyl H atoms and 1.2*U*_eq_ for the remaining H atoms. A rotating group model was used for the methyl groups. The highest residual electron density peak is located at 0.68 Å from C5 and the deepest hole is located at 1.28 Å from C10. A total of 2260 Friedel pairs were merged before final refinement as there is no large anomalous dispersion for the determination of the absolute configuration.

### Crystal data

**Table:**

C_23_H_25_NO_4_	*D*_x_ = 1.348 Mg m^−^^3^
*M**_r_* = 379.44	Melting point = 518–520 K
Orthorhombic, *P*2_1_2_1_2_1_	Mo *K*α radiation, λ = 0.71073 Å
Hall symbol: P 2ac 2ab	Cell parameters from 3142 reflections
*a* = 5.0650 (1) Å	θ = 1.6–30.0°
*b* = 15.0131 (4) Å	µ = 0.09 mm^−^^1^
*c* = 24.5813 (5) Å	*T* = 100 K
*V* = 1869.20 (7) Å^3^	Plate, brown
*Z* = 4	0.40 × 0.21 × 0.04 mm
*F*(000) = 808	

### Data collection

**Table:**

Bruker APEXII CCD area-detector diffractometer	3142 independent reflections
Radiation source: sealed tube	2525 reflections with *I* > 2σ(*I*)
graphite	*R*_int_ = 0.041
φ and ω scans	θ_max_ = 30.0°, θ_min_ = 1.6°
Absorption correction: multi-scan (*SADABS*; Bruker, 2005)	*h* = −6→7
*T*_min_ = 0.964, *T*_max_ = 0.996	*k* = −21→15
17852 measured reflections	*l* = −34→34

### Refinement

**Table:**

Refinement on *F*^2^	Primary atom site location: structure-invariant direct methods
Least-squares matrix: full	Secondary atom site location: difference Fourier map
*R*[*F*^2^ > 2σ(*F*^2^)] = 0.049	Hydrogen site location: inferred from neighbouring sites
*wR*(*F*^2^) = 0.118	H-atom parameters constrained
*S* = 1.03	*w* = 1/[σ^2^(*F*_o_^2^) + (0.0588*P*)^2^ + 0.5624*P*] where *P* = (*F*_o_^2^ + 2*F*_c_^2^)/3
3142 reflections	(Δ/σ)_max_ < 0.001
257 parameters	Δρ_max_ = 0.30 e Å^−^^3^
0 restraints	Δρ_min_ = −0.26 e Å^−^^3^

### Special details

**Table:**

Experimental. The crystal was placed in the cold stream of an Oxford Cryosystems Cobra open-flow nitrogen cryostat (Cosier & Glazer, 1986) operating at 100.0 (1) K.
Geometry. All e.s.d.'s (except the e.s.d. in the dihedral angle between two l.s. planes) are estimated using the full covariance matrix. The cell e.s.d.'s are taken into account individually in the estimation of e.s.d.'s in distances, angles and torsion angles; correlations between e.s.d.'s in cell parameters are only used when they are defined by crystal symmetry. An approximate (isotropic) treatment of cell e.s.d.'s is used for estimating e.s.d.'s involving l.s. planes.
Refinement. Refinement of *F*^2^ against ALL reflections. The weighted *R*-factor *wR* and goodness of fit *S* are based on *F*^2^, conventional *R*-factors *R* are based on *F*, with *F* set to zero for negative *F*^2^. The threshold expression of *F*^2^ > σ(*F*^2^) is used only for calculating *R*-factors(gt) *etc*. and is not relevant to the choice of reflections for refinement. *R*-factors based on *F*^2^ are statistically about twice as large as those based on *F*, and *R*- factors based on ALL data will be even larger.

### Fractional atomic coordinates and isotropic or equivalent isotropic displacement parameters (Å^2^)

**Table:**

	*x*	*y*	*z*	*U*_iso_*/*U*_eq_	
O1	0.3340 (3)	0.33730 (11)	0.16280 (7)	0.0193 (4)	
H1O1	0.4552	0.3307	0.1845	0.029*	
O2	0.7218 (3)	0.37946 (11)	0.22404 (6)	0.0183 (4)	
O3	0.9035 (4)	0.77231 (10)	0.22808 (6)	0.0187 (4)	
H1O3	1.0112	0.8067	0.2415	0.028*	
O4	−0.0692 (3)	0.56738 (12)	0.05943 (7)	0.0220 (4)	
H1O4	−0.1545	0.5244	0.0487	0.026*	
N1	0.6177 (4)	0.64068 (12)	0.18267 (8)	0.0150 (4)	
H1N1	0.6002	0.6956	0.1734	0.018*	
C1	0.1293 (5)	0.44831 (15)	0.11046 (9)	0.0149 (5)	
C2	0.3171 (5)	0.42414 (15)	0.14846 (9)	0.0148 (5)	
C3	0.4888 (4)	0.48744 (14)	0.17270 (9)	0.0137 (4)	
C4	0.6867 (5)	0.46079 (15)	0.21169 (9)	0.0141 (4)	
C5	0.8434 (5)	0.53044 (15)	0.23695 (9)	0.0152 (5)	
C6	1.0356 (5)	0.51065 (15)	0.27656 (9)	0.0193 (5)	
H6A	1.0648	0.4519	0.2868	0.023*	
C7	1.1794 (5)	0.57750 (17)	0.29992 (10)	0.0226 (5)	
H7A	1.3061	0.5639	0.3260	0.027*	
C8	1.1368 (5)	0.66664 (16)	0.28480 (9)	0.0198 (5)	
H8A	1.2342	0.7116	0.3014	0.024*	
C9	0.9534 (5)	0.68805 (15)	0.24586 (9)	0.0159 (5)	
C10	0.8024 (5)	0.61953 (15)	0.22133 (9)	0.0147 (4)	
C11	0.4593 (4)	0.57851 (14)	0.15804 (9)	0.0143 (4)	
C12	0.2707 (5)	0.60543 (15)	0.11958 (9)	0.0149 (5)	
C13	0.1139 (5)	0.53968 (15)	0.09674 (9)	0.0167 (5)	
C14	0.2242 (5)	0.70302 (15)	0.10646 (10)	0.0176 (5)	
H14A	0.0747	0.7072	0.0819	0.021*	
H14B	0.1765	0.7336	0.1398	0.021*	
C15	0.4547 (5)	0.75103 (16)	0.08107 (9)	0.0185 (5)	
H15A	0.5447	0.7210	0.0537	0.022*	
C16	0.5433 (5)	0.83205 (16)	0.09377 (10)	0.0214 (5)	
C17	0.7643 (6)	0.87496 (19)	0.06275 (13)	0.0337 (7)	
H17A	0.8238	0.8353	0.0347	0.051*	
H17B	0.9077	0.8877	0.0871	0.051*	
H17C	0.7028	0.9294	0.0466	0.051*	
C18	0.4340 (6)	0.88750 (17)	0.13926 (11)	0.0284 (6)	
H18A	0.2903	0.8564	0.1562	0.043*	
H18B	0.3717	0.9432	0.1250	0.043*	
H18C	0.5700	0.8984	0.1656	0.043*	
C19	−0.0597 (5)	0.38065 (15)	0.08666 (9)	0.0179 (5)	
H19A	−0.0638	0.3287	0.1101	0.022*	
H19B	−0.2358	0.4060	0.0862	0.022*	
C20	0.0113 (5)	0.35141 (15)	0.02980 (9)	0.0190 (5)	
H20A	0.1620	0.3164	0.0266	0.023*	
C21	−0.1154 (5)	0.36960 (16)	−0.01649 (10)	0.0219 (5)	
C22	−0.0143 (7)	0.3355 (2)	−0.07035 (10)	0.0323 (7)	
H22A	0.1369	0.2982	−0.0643	0.049*	
H22B	0.0349	0.3850	−0.0929	0.049*	
H22C	−0.1502	0.3017	−0.0880	0.049*	
C23	−0.3584 (6)	0.4259 (2)	−0.02118 (12)	0.0333 (6)	
H23A	−0.4316	0.4356	0.0143	0.050*	
H23B	−0.4859	0.3961	−0.0436	0.050*	
H23C	−0.3134	0.4821	−0.0373	0.050*	

### Atomic displacement parameters (Å^2^)

**Table:**

	*U*^11^	*U*^22^	*U*^33^	*U*^12^	*U*^13^	*U*^23^
O1	0.0209 (8)	0.0137 (8)	0.0235 (8)	−0.0020 (7)	−0.0046 (7)	0.0007 (7)
O2	0.0205 (8)	0.0100 (7)	0.0243 (8)	−0.0001 (7)	−0.0030 (7)	0.0012 (7)
O3	0.0236 (9)	0.0098 (7)	0.0227 (8)	−0.0018 (7)	−0.0055 (7)	−0.0001 (6)
O4	0.0206 (8)	0.0205 (8)	0.0251 (9)	−0.0028 (8)	−0.0084 (7)	0.0007 (7)
N1	0.0153 (9)	0.0083 (9)	0.0214 (9)	0.0004 (8)	−0.0029 (8)	0.0009 (7)
C1	0.0155 (10)	0.0137 (10)	0.0155 (10)	−0.0026 (9)	0.0010 (9)	−0.0029 (9)
C2	0.0167 (11)	0.0107 (10)	0.0169 (11)	−0.0015 (9)	0.0031 (9)	−0.0020 (9)
C3	0.0143 (10)	0.0110 (9)	0.0158 (10)	−0.0010 (9)	0.0004 (9)	−0.0014 (8)
C4	0.0147 (10)	0.0133 (10)	0.0141 (10)	0.0009 (9)	−0.0006 (9)	−0.0013 (9)
C5	0.0169 (11)	0.0121 (10)	0.0164 (10)	−0.0003 (9)	0.0003 (9)	−0.0013 (9)
C6	0.0255 (12)	0.0112 (10)	0.0213 (12)	0.0022 (10)	−0.0066 (10)	0.0013 (9)
C7	0.0264 (13)	0.0179 (11)	0.0236 (12)	0.0013 (11)	−0.0108 (11)	0.0011 (10)
C8	0.0246 (12)	0.0135 (11)	0.0211 (12)	−0.0040 (10)	−0.0063 (10)	−0.0031 (9)
C9	0.0194 (11)	0.0113 (10)	0.0170 (11)	−0.0017 (9)	0.0008 (10)	−0.0021 (9)
C10	0.0152 (10)	0.0144 (10)	0.0145 (10)	0.0006 (9)	0.0003 (9)	−0.0003 (9)
C11	0.0125 (10)	0.0115 (9)	0.0190 (11)	−0.0019 (9)	0.0013 (9)	−0.0006 (9)
C12	0.0161 (10)	0.0099 (10)	0.0186 (11)	0.0000 (9)	−0.0008 (9)	0.0011 (9)
C13	0.0159 (11)	0.0176 (11)	0.0167 (10)	0.0012 (10)	0.0006 (9)	0.0014 (9)
C14	0.0154 (11)	0.0121 (10)	0.0252 (12)	−0.0004 (9)	−0.0036 (10)	0.0024 (9)
C15	0.0200 (11)	0.0174 (11)	0.0181 (11)	0.0026 (10)	−0.0021 (9)	0.0023 (9)
C16	0.0192 (11)	0.0191 (11)	0.0258 (12)	−0.0022 (10)	−0.0084 (10)	0.0073 (10)
C17	0.0237 (13)	0.0263 (14)	0.0510 (18)	−0.0056 (13)	−0.0017 (14)	0.0111 (13)
C18	0.0376 (15)	0.0150 (11)	0.0326 (14)	−0.0031 (12)	−0.0083 (13)	−0.0037 (11)
C19	0.0172 (11)	0.0155 (10)	0.0211 (11)	−0.0045 (10)	−0.0004 (9)	0.0005 (9)
C20	0.0202 (11)	0.0148 (10)	0.0220 (12)	−0.0034 (10)	0.0012 (10)	−0.0033 (9)
C21	0.0283 (13)	0.0170 (11)	0.0205 (12)	−0.0085 (11)	0.0017 (11)	−0.0007 (10)
C22	0.0453 (17)	0.0313 (14)	0.0204 (13)	−0.0074 (15)	0.0010 (13)	−0.0022 (11)
C23	0.0268 (14)	0.0446 (17)	0.0287 (14)	−0.0006 (14)	−0.0086 (12)	0.0017 (13)

### Geometric parameters (Å, °)

**Table:**

O1—C2	1.353 (3)	C12—C14	1.519 (3)
O1—H1O1	0.8200	C14—C15	1.508 (3)
O2—C4	1.271 (3)	C14—H14A	0.9700
O3—C9	1.362 (3)	C14—H14B	0.9700
O3—H1O3	0.8200	C15—C16	1.334 (3)
O4—C13	1.369 (3)	C15—H15A	0.9300
O4—H1O4	0.8200	C16—C17	1.500 (4)
N1—C10	1.371 (3)	C16—C18	1.500 (4)
N1—C11	1.372 (3)	C17—H17A	0.9600
N1—H1N1	0.8600	C17—H17B	0.9600
C1—C2	1.381 (3)	C17—H17C	0.9600
C1—C13	1.415 (3)	C18—H18A	0.9600
C1—C19	1.514 (3)	C18—H18B	0.9600
C2—C3	1.419 (3)	C18—H18C	0.9600
C3—C11	1.422 (3)	C19—C20	1.508 (3)
C3—C4	1.443 (3)	C19—H19A	0.9700
C4—C5	1.452 (3)	C19—H19B	0.9700
C5—C10	1.407 (3)	C20—C21	1.335 (3)
C5—C6	1.408 (3)	C20—H20A	0.9300
C6—C7	1.366 (3)	C21—C23	1.497 (4)
C6—H6A	0.9300	C21—C22	1.509 (3)
C7—C8	1.406 (3)	C22—H22A	0.9600
C7—H7A	0.9300	C22—H22B	0.9600
C8—C9	1.372 (3)	C22—H22C	0.9600
C8—H8A	0.9300	C23—H23A	0.9600
C9—C10	1.417 (3)	C23—H23B	0.9600
C11—C12	1.404 (3)	C23—H23C	0.9600
C12—C13	1.386 (3)		
			
C2—O1—H1O1	109.5	C12—C14—H14A	108.4
C9—O3—H1O3	109.5	C15—C14—H14B	108.4
C13—O4—H1O4	109.5	C12—C14—H14B	108.4
C10—N1—C11	123.20 (19)	H14A—C14—H14B	107.5
C10—N1—H1N1	118.4	C16—C15—C14	126.8 (2)
C11—N1—H1N1	118.4	C16—C15—H15A	116.6
C2—C1—C13	117.0 (2)	C14—C15—H15A	116.6
C2—C1—C19	121.4 (2)	C15—C16—C17	121.6 (3)
C13—C1—C19	121.6 (2)	C15—C16—C18	123.8 (2)
O1—C2—C1	118.2 (2)	C17—C16—C18	114.6 (2)
O1—C2—C3	119.8 (2)	C16—C17—H17A	109.5
C1—C2—C3	122.0 (2)	C16—C17—H17B	109.5
C2—C3—C11	118.2 (2)	H17A—C17—H17B	109.5
C2—C3—C4	121.2 (2)	C16—C17—H17C	109.5
C11—C3—C4	120.5 (2)	H17A—C17—H17C	109.5
O2—C4—C3	121.5 (2)	H17B—C17—H17C	109.5
O2—C4—C5	120.9 (2)	C16—C18—H18A	109.5
C3—C4—C5	117.64 (19)	C16—C18—H18B	109.5
C10—C5—C6	119.4 (2)	H18A—C18—H18B	109.5
C10—C5—C4	119.1 (2)	C16—C18—H18C	109.5
C6—C5—C4	121.4 (2)	H18A—C18—H18C	109.5
C7—C6—C5	120.3 (2)	H18B—C18—H18C	109.5
C7—C6—H6A	119.9	C20—C19—C1	113.73 (19)
C5—C6—H6A	119.9	C20—C19—H19A	108.8
C6—C7—C8	120.4 (2)	C1—C19—H19A	108.8
C6—C7—H7A	119.8	C20—C19—H19B	108.8
C8—C7—H7A	119.8	C1—C19—H19B	108.8
C9—C8—C7	120.8 (2)	H19A—C19—H19B	107.7
C9—C8—H8A	119.6	C21—C20—C19	128.0 (2)
C7—C8—H8A	119.6	C21—C20—H20A	116.0
O3—C9—C8	124.5 (2)	C19—C20—H20A	116.0
O3—C9—C10	116.0 (2)	C20—C21—C23	125.2 (2)
C8—C9—C10	119.5 (2)	C20—C21—C22	121.0 (2)
N1—C10—C5	120.7 (2)	C23—C21—C22	113.8 (2)
N1—C10—C9	119.7 (2)	C21—C22—H22A	109.5
C5—C10—C9	119.6 (2)	C21—C22—H22B	109.5
N1—C11—C12	119.97 (19)	H22A—C22—H22B	109.5
N1—C11—C3	118.7 (2)	C21—C22—H22C	109.5
C12—C11—C3	121.3 (2)	H22A—C22—H22C	109.5
C13—C12—C11	117.3 (2)	H22B—C22—H22C	109.5
C13—C12—C14	120.8 (2)	C21—C23—H23A	109.5
C11—C12—C14	121.8 (2)	C21—C23—H23B	109.5
O4—C13—C12	116.3 (2)	H23A—C23—H23B	109.5
O4—C13—C1	119.5 (2)	C21—C23—H23C	109.5
C12—C13—C1	124.2 (2)	H23A—C23—H23C	109.5
C15—C14—C12	115.4 (2)	H23B—C23—H23C	109.5
C15—C14—H14A	108.4		
			
C13—C1—C2—O1	179.2 (2)	O3—C9—C10—C5	179.3 (2)
C19—C1—C2—O1	2.1 (3)	C8—C9—C10—C5	−0.4 (3)
C13—C1—C2—C3	−0.3 (3)	C10—N1—C11—C12	178.7 (2)
C19—C1—C2—C3	−177.5 (2)	C10—N1—C11—C3	−0.6 (3)
O1—C2—C3—C11	−177.9 (2)	C2—C3—C11—N1	177.7 (2)
C1—C2—C3—C11	1.7 (3)	C4—C3—C11—N1	−1.7 (3)
O1—C2—C3—C4	1.4 (3)	C2—C3—C11—C12	−1.7 (3)
C1—C2—C3—C4	−179.0 (2)	C4—C3—C11—C12	179.0 (2)
C2—C3—C4—O2	3.0 (3)	N1—C11—C12—C13	−179.0 (2)
C11—C3—C4—O2	−177.7 (2)	C3—C11—C12—C13	0.3 (3)
C2—C3—C4—C5	−176.4 (2)	N1—C11—C12—C14	−3.6 (3)
C11—C3—C4—C5	2.9 (3)	C3—C11—C12—C14	175.7 (2)
O2—C4—C5—C10	178.7 (2)	C11—C12—C13—O4	179.85 (19)
C3—C4—C5—C10	−2.0 (3)	C14—C12—C13—O4	4.4 (3)
O2—C4—C5—C6	−1.2 (3)	C11—C12—C13—C1	1.2 (3)
C3—C4—C5—C6	178.2 (2)	C14—C12—C13—C1	−174.3 (2)
C10—C5—C6—C7	0.5 (4)	C2—C1—C13—O4	−179.8 (2)
C4—C5—C6—C7	−179.6 (2)	C19—C1—C13—O4	−2.7 (3)
C5—C6—C7—C8	0.1 (4)	C2—C1—C13—C12	−1.2 (3)
C6—C7—C8—C9	−0.9 (4)	C19—C1—C13—C12	176.0 (2)
C7—C8—C9—O3	−178.7 (2)	C13—C12—C14—C15	−120.8 (2)
C7—C8—C9—C10	1.0 (4)	C11—C12—C14—C15	64.0 (3)
C11—N1—C10—C5	1.6 (3)	C12—C14—C15—C16	−136.1 (2)
C11—N1—C10—C9	−178.3 (2)	C14—C15—C16—C17	−175.8 (2)
C6—C5—C10—N1	179.6 (2)	C14—C15—C16—C18	4.2 (4)
C4—C5—C10—N1	−0.2 (3)	C2—C1—C19—C20	−103.0 (3)
C6—C5—C10—C9	−0.4 (3)	C13—C1—C19—C20	79.9 (3)
C4—C5—C10—C9	179.7 (2)	C1—C19—C20—C21	−110.2 (3)
O3—C9—C10—N1	−0.7 (3)	C19—C20—C21—C23	1.2 (4)
C8—C9—C10—N1	179.6 (2)	C19—C20—C21—C22	179.3 (2)

### Hydrogen-bond geometry (Å, °)

**Table:**

*D*—H···*A*	*D*—H	H···*A*	*D*···*A*	*D*—H···*A*
O1—H1O1···O2	0.82	1.82	2.554 (2)	149
O3—H1O3···O2^i^	0.82	1.93	2.752 (2)	175
N1—H1N1···O3	0.86	2.34	2.692 (3)	105
C14—H14A···O4	0.97	2.29	2.773 (3)	110
C19—H19A···O1	0.97	2.40	2.811 (3)	105

Symmetry codes:  (i) −*x*+2, *y*+1/2, −*z*+1/2.
